# Correction: Evaluating the foundations that help avert antimicrobial resistance: Performance of essential water sanitation and hygiene functions in hospitals and requirements for action in Kenya

**DOI:** 10.1371/journal.pone.0228489

**Published:** 2020-01-24

**Authors:** The PLOS ONE Staff

## Notice of republication

An incorrect grant number was included in the XML of this article. The article was republished on January 13, 2020, to correct for this error. The publisher apologizes for this error.
