# Supplementary material for: Evaluating the foundations that help avert antimicrobial resistance: Performance of essential water sanitation and hygiene functions in hospitals and requirements for action in Kenya
Source: PLoS One. 2019 Oct 9;14(10):e0222922. doi: 10.1371/journal.pone.0222922 (PMC6785173; doi:10.1371/journal.pone.0222922)
Supplement: S1 Table — (DOCX) [file pone.0222922.s001.docx]

**S1 Table Aggregate Ward Indicator Scores**

| **DOMAIN** | **Indicator Description** | **Minimum Score** | **lower IQR** | **Median Score** | **Upper IQR** | **Maximum Score** | **Mean Score** |
| --- | --- | --- | --- | --- | --- | --- | --- |
| **Water** | Showers available | 58.33 | 75.00 | 84.82 | 92.86 | 100 | 83.62 |
|  | Shower lighting present | 16.67 | 44.18 | 60.63 | 69.17 | 96.43 | 56.99 |
|  | Water Services Available in quantity | 63.64 | 92.71 | 100 | 100 | 100 | 93.12 |
|  | Drinking water Station | 0 | 0 | 17.14 | 72.57 | 100 | 34.05 |
|  | Drinking Water Storage | 0 | 0 | 3.13 | 13.49 | 28.57 | 7.96 |
|  | End points(taps) connected | 0 | 46.88 | 54.91 | 78.75 | 91.67 | 58.19 |
| **Sanitation** | Waste collection bins | 50 | 50 | 57.74 | 66.25 | 100 | 62.92 |
|  | Waste segregation | 0 | 12.50 | 50 | 72.92 | 94.44 | 45.32 |
|  | Waste management SOP | 3.57 | 13.54 | 21.88 | 42.56 | 80 | 29.98 |
|  | Waste handling PPE | 28.57 | 50 | 60.42 | 71.67 | 100 | 60.99 |
|  | Toilets available in correct numbers | 55.00 | 66.67 | 71.43 | 88.33 | 100 | 76.23 |
|  | Toilets well lit | 16.67 | 37.20 | 58.12 | 65.00 | 91.67 | 54.48 |
|  | Toilets separate (Male/ Female, Staff/Patients) | 42.86 | 74.55 | 92.92 | 100 | 100 | 84.73 |
|  | Toilet with Menstrual Hygiene services | 20 | 64.88 | 83.33 | 89.38 | 100 | 73.28 |
|  | Toilets for reduced Mobility | 0 | 0 | 0 | 4.69 | 33.33 | 4.91 |
|  | Toilet Hand wash station | 28.57 | 42.86 | 50 | 53.13 | 75.00 | 49.20 |
|  | Toilet cleaning record | 0 | 0 | 0 | 50 | 95.00 | 26.64 |
| **Hygiene** | Insecticide treated nets on beds | 0 | 0 | 24.70 | 54.69 | 83.33 | 29.51 |
|  | Mechanism for IPC supplies tracking | 0 | 17.86 | 81.94 | 100 | 100 | 63.41 |
|  | Cleaning records available | 0 | 0 | 0 | 0 | 90 | 14.52 |
|  | Natural ventilation available | 65.00 | 94.27 | 100 | 100 | 100 | 95.14 |
|  | 2.5 Metre bed distance | 0 | 0 | 4.17 | 18.85 | 41.67 | 11.31 |
|  | General lighting adequate | 58.33 | 95.55 | 100 | 100 | 100 | 95.44 |
|  | Clean floors/surfaces | 50 | 70.31 | 87.50 | 100 | 100 | 83.79 |
|  | cleaning materials available | 41.67 | 50 | 56.70 | 64.58 | 83.33 | 58.12 |
|  | Hand Hygiene-Points of care | 35.71 | 50 | 51.79 | 56.08 | 70 | 53.35 |
|  | Hand Hygiene Promotion materials | 0 | 33.33 | 43.30 | 50 | 90 | 41.67 |
|  | Hand Hygiene-Service areas | 18.75 | 50 | 50 | 50 | 77.78 | 50.88 |
|  | Hand Hygiene compliance activities | 0 | 0 | 45.00 | 53.41 | 80 | 34.99 |
| **Organisation Management** | Staff rewards for good performance | 0 | 0 | 26.70 | 50 | 61.11 | 25.78 |
|  | Ward based audits | 0 | 21.97 | 36.61 | 48.96 | 70 | 35.28 |
|  | New staff orientation | 43.75 | 78.33 | 92.26 | 100 | 100 | 87.06 |
|  | Staff IPC training | 0 | 19.32 | 35.83 | 50 | 100 | 37.89 |
|  | IPC Focal person | 0 | 2.08 | 20.83 | 48.44 | 91.67 | 31.88 |
