# Supplementary material for: Evaluating the foundations that help avert antimicrobial resistance: Performance of essential water sanitation and hygiene functions in hospitals and requirements for action in Kenya
Source: PLoS One. 2019 Oct 9;14(10):e0222922. doi: 10.1371/journal.pone.0222922 (PMC6785173; doi:10.1371/journal.pone.0222922)
Supplement: S1 File — (DOCX) [file pone.0222922.s002.docx]

**S1 File. WASH Data collection and Standard Operating procedures tool**

**WASH ASSESSMENT BOOKLET**

**WASH TEAM LIST**

**List all persons that have contributed to the assessment or have participated in briefings/discussions during the WASH baseline assessment, each day**

| **Date** | **Name** | **Job Title / Role** | **Role / Involvement During**  **WASH Baseline Assessment** | **Contact Details (e.g. phone**  **Number and e-mail if available)** |
| --- | --- | --- | --- | --- |
|  |  |  |  |  |
|  |  |  |  |  |
|  |  |  |  |  |
|  |  |  |  |  |

**Additional Comments on Roles and Contributions during WASH Baseline Assessment**

**Notes from Initial Briefing**

**During the initial briefing, you must hand in appropriate approval letters, explain the purpose of the survey in the context of the broader study, explain what the assessment will involve, and when and how feed-back will be provided. You must explain that the assessment will involve taking pictures, to assist with the scoring of indicators in consultation with the research teams operating in other hospitals. Pictures will NOT include patients or staff, or signs that can identify the hospital. They will be kept in anonymised format and encrypted and will be destroyed as soon as the indicators are unequivocally scored. ONLY THE THREE WASH TEAM SUPERVISORS (i.e. NOT the team leaders) ARE ALLOWED TO TAKE PICTURES. During the briefing, take the opportunity to ask questions to help plan your visit: ask about numbers of wards, names of the wards, structure of the hospital, and permanent and/or temporary local WASH and/or IPC improvement activities by NGOs or other, which may affect the interpretation of your findings. Ask questions around hospital demographics and capacity, such as total number of beds (i.e. hospital capacity), numbers of outpatients on a typical day, average number of surgical procedures daily or weekly, total number of admissions daily or weekly, as well of average number of births in a day or week. You must write down the notes from the meeting in the sections below. If you gather relevant details in subsequent days, which refer to hospital structure, capacity and demographics, add the information here as well.**

**WARD-LEVEL**

**ASSESSMENT FORMS**

**Explanatory Notes for Ward-Level WASH Indicators**

| **WATER** | | |
| --- | --- | --- |
| **1.2** | | For an intermittent piped-water supply, e.g. available 8 hours per day. |
| **1.3** | | Describe if drinking stations are provided with railings and a seat. |
| **1.6** | | Describe how many sinks have functioning pipes for water coming in and going out and how many sinks have functioning pipes for water coming in only. |
| **SANITATION** | | |
| **2.1** | | Improved sanitation facilities include flush toilets into managed sewer or septic tank and soakaway pit, VIP latrines, pit latrines with slab, and composting toilets.  To be considered usable, a toilet/latrine should have a door which is unlocked when not in use (or for which a key is available at any time) and can be locked from the inside during use, there should be no major holes in the structure, the hole or pit should not be blocked, water should be available for flush/pour flush toilets, and there should be no cracks, or leaks in the toilet structure. It should be within the grounds of the facility and it should be clean as noted by absence of waste, visible dirt and excreta and insects. |
| **2.4** | | Toilets should have a bin for disposal of waste or an area for washing, with water available. |
| **2.5** | | A toilet can be considered to meet the needs of people with reduced mobility if it meets the following conditions: can be accessed without stairs or steps, handrails for support are attached either to the floor or sidewalls, the door is at least 80 cm wide, the toilet has a raised seat (between 40-48cm from the floor), a backrest and the cubicle has space for circulation/manoeuvring (150x150 cm). The sink tap, and water outside should also be accessible and the top of the sink 75cm from the floor (with knee clearance). Switches for lights, where relevant, should also be at an accessible height (max 120 cm). All specifications are based on ISO 21542:2011 – Building construction - Accessibility and usability of the built environment, available at: http://www.iso.org/iso/home/store/catalogue_tc/catalogue_detail.htm?csnumber=50498 |
| **2.6** | | A functional hand hygiene station may consist of soap and water with a basin/pan for washing hands. Water should not be chlorinated. Alcohol-based hand rub is *not* suitable for use at latrines. |
| **2.7** | | For low literacy or illiterate cleaners, this should be adapted and simplified accordingly with recognizable pictures and illustrations. |
| **2.10** | | Lighting for latrines is necessary in all facilities where night-time services are provided and where there is not sufficient natural light to safely use the latrine during the day. |
| **HEALTHCARE WASTE** | | |
| **2.12** | | Functional means containers should not be ¾ full, be leak-proof with a lid and clearly labelled (i.e. easily distinguishable according to a colour, label or symbol). |
| **2.22** | | Protective equipment for people handling waste management includes: gloves, apron and tough rubber boots. |
| **HAND HYGIENE** | | |
| **3.1** | | Point of care is where three elements come together: the patient, the health-care workers and care or treatment involving contact with the patient or his/her surroundings. This may include consultation rooms, operating rooms, delivery rooms AND laboratory. Hand hygiene stations should have a sink or bucket with tap and water with soap OR alcohol-based hand-rub. **There should be at least two hand hygiene stations in a ward with more than 20 beds.** Verify that water is available from the tap. |
| **3.2** | | Key places include at points of care and within 5 m of latrines. You may want to write down where stations are located (and were they are missing) in your comments box. |
| **3.3** | | Sink or bucket with tap and water with soap OR alcohol-based hand-rub. Service areas include sterilization room (where applicable), laboratory (where applicable) and showers. (Toilets are included under 2.7) |
| **3.4** | | Tap and water with soap. |
| **ENVIRONMENT, CLEANLINESS AND DISINFECTION** | | |
| **3.8** | Clean as noted by absence of waste, visible dirt and excreta and insects. Environmental surfaces or objects contaminated with blood, other body fluids, secretions or excretions are cleaned and disinfected as soon as possible using standard hospital detergents/disinfectants. | |
| **MANAGEMENT** | | |
| **N/A** | N/A | |

**Ward Assessment - Notes**

**Before you begin your assessment and scoring, take the time to walk through the ward and observe all the details. Speak to staff, take pictures where needed (no patients/staff/hospital identifying signs allowed in pictures. ONLY THE THREE WASH TEAM SUPERVISORS (i.e. NOT the team leaders) ARE ALLOWED TO TAKE PICTURES), count numbers of functional toilets, beds, sinks, bulbs, hand washing stations, soap dispensers, etc. Inspect bins, cleaning materials, etc. Write notes in the space below. Sit with the team and try to score some of the indicators. If needed walk through the ward again to gather mode details which will allow you to fine-tune the scoring. In the process, write down all your questions. You can then address all your queries with a member of staff in one go. This should allow you to complete the assessment.**

**ENTER WARD NAME: _____________________________**

| **Ward Name: ________________________**  **Facility Code:________________________** | | **BASELINE ASSESSMENT**  **(Clearly tick the box that best describes your findings)** | | | **Assessment Date:** |
| --- | --- | --- | --- | --- | --- |
| **1** | **WATER** | **Meets**  **Target**  **(+++)** | **Partially Meets Target**  **(++)** | **Does not**  **Meet Target**  **(+)** | **Explain the reasoning for your scoring and comment as appropriate. For indicators with an asterisk, read the notes before scoring.** |
| **1.2*** | Water services available at all times and of sufficient quantity for all uses (refers to a typical average week). | **□** Yes, every day and of sufficient quantity | **□** More than 5 days per week or every day but not sufficient quantity | **□** Fewer than 5 days per week |  |
| **1.3*** | A reliable drinking water station is present and accessible for staff, patients and carers at all times. | **□** Yes, at all times and accessible to all | **□** Sometimes, or not available for all users | **□** Not available |  |
| **1.4** | Drinking water is safely stored in a clean bucket/ tank with cover and tap. | **□** Yes | **□** Only some drinking water points are safely stored | **□** Not safely stored in any water points or no drinking water available |  |
| **1.6*** | All end points (i.e. taps) are connected to an available and functioning water supply. | **□** Yes, all are connected and functioning | **□** More than half of all endpoints are connected and functioning | **□** No, less than half of all endpoints connected and functioning |  |
| **1.13** | At least one shower or bathing area is available per 40 patients in inpatient settings and is functioning and accessible. | **□** Yes | **□** Showers available, but no water or in disrepair or showers available but fewer than1 per 40 | **□** No showers. |  |
| **1.14** | Shower(s) are adequately lit, including at night. | **□** Yes | **□** Lighting infrastructure exists, but not functioning | **□** Not adequately lit or no lighting infrastructure |  |

| **Ward Name: ________________________**  **Facility Code:________________________** | | **BASELINE ASSESSMENT**  **(Clearly tick the box that best describes your findings)** | | | **Assessment Date:** |
| --- | --- | --- | --- | --- | --- |
| **2.a** | **SANITATION** | **Meets**  **Target**  **(+++)** | **Partially Meets Target**  **(++)** | **Does not**  **Meet Target**  **(+)** | **Explain the reasoning for your scoring and comment as appropriate. For indicators with an asterisk, read the notes before scoring.** |
| **2.1*** | Number of available and usable toilets or improved latrines for patients. | **□** One for every 20 inpatients. | **□** Sufficient number present but not all functioning or insufficient number. | **□** Less than 50% of required number of latrines available and functioning. |  |
| **2.2** | Toilets or improved latrines in the ward clearly separated for staff and patients | **□** Yes. | N/A | **□** No separate latrines. |  |
| **2.4*** | At least one toilet or improved latrine provides the means to manage menstrual hygiene needs. | **□** Yes. | **□** Yes, but toilet is not clean or in disrepair. | **□** No. | (Score this question in female wards only) |
| **2.5*** | At least one toilet in the ward meets the needs of people with reduced mobility. | **□** Yes. | **□** Yes, but not available or in disrepair. | **□** No toilet for disabled users. |  |
| **2.6*** | Functioning hand hygiene stations within 5 m of latrines. | **□** Yes. | **□** Present, not functioning or no water or soap. | **□** Not present. |  |
| **2.7*** | Record of cleaning visible and signed by the cleaners each day. | **□** Yes. | **□** Toilets cleaned but not recorded. | **□** No record / toilets cleaned less than once a day. |  |
| **2.10*** | Latrines are adequately lit, including at night. | **□** Yes | **□** Lighting infrastructure exists, but not functioning. | **□** Not adequately lit or no lighting infrastructure. |  |

| **Ward Name: ________________________**  **Facility Code:________________________** | | **BASELINE ASSESSMENT**  **(Clearly tick the box that best describes your findings)** | | | **Assessment Date:** |
| --- | --- | --- | --- | --- | --- |
| **2.b** | **HEALTHCARE WASTE** | **Meets**  **Target**  **(+++)** | **Partially Meets Target**  **(++)** | **Does not**  **Meet Target**  **(+)** | **Explain the reasoning for your scoring and comment as appropriate. For indicators with an asterisk, read the notes before scoring.** |
| **2.12*** | Functional waste collection containers for 1) non-infectious (general) waste, 2) infectious waste and 3) sharps waste in close proximity to all waste generation points. | **□** Yes. | **□** Separate bins present but lids missing or more than ¾ full; only two bins (instead of three); or at some but not all waste generation points. | **□** No bins or separate sharps disposal. |  |
| **2.13** | Waste correctly segregated at all waste generation points. | **□** Yes. | **□** Some sorting but not all correctly or not practiced throughout the ward. | **□** No sorting. |  |
| **2.21** | Protocol or SOP (Standard Operating Procedure) for safe management of healthcare waste clearly visible and legible. | **□** Yes, visible and implemented. | **□** Written but not visible or implemented. | **□** No protocol/SOP in place. |  |
| **2.22*** | Appropriate protective equipment for all staff in charge of waste disposal. | **□** Yes. | **□** Some equipment available, but not for all staff, or available but damaged. | **□** None available. |  |

| **Ward Name: ________________________**  **Facility Code:________________________** | | **BASELINE ASSESSMENT**  **(Clearly tick the box that best describes your findings)** | | | **Assessment Date:** |
| --- | --- | --- | --- | --- | --- |
| **3.a** | **HAND HYGIENE** | **Meets**  **Target**  **(+++)** | **Partially Meets Target**  **(++)** | **Does not**  **Meet Target**  **(+)** | **Explain the reasoning for your scoring and comment as appropriate. For indicators with an asterisk, read the notes before scoring.** |
| **3.1*** | Functioning hand hygiene stations are available at all points of care. | **□** Yes. | **□** Stations present, but no water and/or soap or alcohol hand-rub solution. | **□** Not present. |  |
| **3.2*** | Hand hygiene promotion materials clearly visible and understandable at key places. | **□** Yes. | **□** Some places but not all. | **□** None. |  |
| **3.3*** | Functioning hand hygiene stations are available in-service areas. | **□** Yes. | **□** Stations present, but no water and/or soap or alcohol hand-rub solution. | **□** Not present. |  |
| **3.5** | Hand hygiene compliance activities are undertaken regularly. | **□** Yes. | **□** Compliance activities in policy, but not carried out with any regularity. | **□** No compliance activities. |  |

| **Ward Name: ________________________**  **Facility Code:________________________** | | **BASELINE ASSESSMENT**  **(Clearly tick the box that best describes your findings)** | | | **Assessment Date:** |
| --- | --- | --- | --- | --- | --- |
| **3.b** | **ENVIRONMENT, CLEANLINESS AND DISINFECTION** | **Meets**  **Target**  **(+++)** | **Partially Meets Target**  **(++)** | **Does not**  **Meet Target**  **(+)** | **Explain the reasoning for your scoring and comment as appropriate. For indicators with an asterisk, read the notes before scoring.** |
| **3.7** | General lighting sufficiently powered and adequate to ensure safe provision of healthcare including at night. | **□** Yes, always. | **□** Yes, sometimes. | **□** Never. |  |
| **3.8*** | Floors and horizontal work surfaces appear clean. | **□** Yes. | **□** Some floors and work surfaces appear clean, but others do not. | **□** Most and/or all floors and surfaces are visibly dirty. |  |
| **3.9** | Appropriate and well-maintained materials for cleaning (i.e. detergent, mops, buckets, etc.) are available. | **□** Yes. | **□** Yes, available but not well maintained. | **□** No materials available. |  |
| **3.12** | Beds have insecticide treated nets to protect patients from mosquito-borne diseases. | **□** Yes, on all beds. | **□** Available on some but not all beds, or available but with rips and or holes. | **□** No bed nets available. |  |
| **3.13** | A mechanism exists within the ward to track supply of IPC-related materials (such as gloves and protective equipment) to identify stock-outs. | **□** Yes. | **□** Mechanism exists but is not enforced. | **□** No mechanism exists. |  |
| **3.14** | Record of cleaning visible and signed by the cleaners each day. | **□** Yes. | **□** Record exists but is not completed daily or is outdated. | **□** No record of floors and surfaces being cleaned. |  |
| **3.16** | The facility has sufficient natural ventilation and where the climate allows, large opening windows, skylights and other vents to optimize natural ventilation. | **□** Yes. | **□** Some ventilation but not well maintained or insufficient to produce natural ventilation. | **□** No. |  |
| **3.18** | **□** Beds for patients should be separated by a distance of 2.5 metres from the centre of one bed to the other and each bed has only one patient. | **□** Yes, all beds meet this guidance. | **□** Some but not all beds fit this criteria. | **□** No beds meet this criteria. |  |

| **Ward Name: ________________________**  **Facility Code:________________________** | | **BASELINE ASSESSMENT**  **(Clearly tick the box that best describes your findings)** | | | **Assessment Date:** |
| --- | --- | --- | --- | --- | --- |
| **4** | **MANAGEMENT** | **Meets**  **Target**  **(+++)** | **Partially Meets Target**  **(++)** | **Does not**  **Meet Target**  **(+)** | **Explain the reasoning for your scoring and comment as appropriate. For indicators with an asterisk, read the notes before scoring.** |
| **4.6** | Regular ward-based audits by external facility personnel (not ward staff) are undertaken to assess the availability of hand-rub, soap, single use towels and other hand hygiene resources. | **□** Yes. | **□** Undertaken less than once a week or assessment is incomplete. | **□** Not undertaken. |  |
| **4.7** | New healthcare personnel receive IPC training as part of their orientation program. | **□** Yes. | **□** Some but not all staff. | **□** No training. |  |
| **4.8** | Healthcare staff are trained on WASH/IPC each year. | **□** Yes. | **□** Staff are trained but not every year or only some staff are trained. | **□** No training. |  |
| **4.9** | The ward has a WASH or IPC focal person. | **□** Yes. | **□** Yes, but focal point does not have sufficient time, resources or motivation to carry out duties. | **□** No. |  |
| **4.11** | High performing staff in the ward are recognized and rewarded and those that do not perform are dealt with accordingly. | **□** Yes. | **□** Either high or low performers addressed but not both. | **□** No action or recognition of staff based on performance. |  |

**FACILITY**

**ASSESSMENT FORMS**

**Explanatory Notes for Facility-Level WASH Indicators**

| **WATER** | |  |
| --- | --- | --- |
| **1.1** | Improved water sources in healthcare settings include piped water, boreholes/tube wells, protected wells, protected springs, rainwater, and packaged or delivered water.  This refers to the water supply for general purposes, including drinking, washing and cleaning. |  |
| **1.2** | For an intermittent piped-water supply, e.g. available 8 hours per day. |  |
| **1.3** | Describe if drinking water stations are provided with railings and a seat. |  |
| **1.8** | Water needs will vary depending on the type of facility and number of patients. To calculate the facility’s water requirements, add up the following requirements (source: WHO 2008 *Essential environmental standards in healthcare*) or applicable national standards.  **Outpatients** (5 litres/consultation) + **Inpatients** (40–60 litres/patient/day) + **Operating theatre or maternity unit** (100 litres/intervention) + **Dry or supplementary feeding centre** (0.5–5 litres/consultation depending on waiting time) + **Cholera treatment centre** (60 litres/patient/day).  Acceptable storage methods include: clean, covered and well-maintained containers which prevent contamination from entering and are free from any cracks, leaks, etc. Such containers should also allow for water to be extracted without hands or other potentially contaminated surfaces from touching the water (i.e. through use of a tap). |  |
| **1.9** | Such technologies should meet one of WHO’s Household Water Treatment and Safe Storage (HWTS) performance categories and generally involve filters, boiling, solar, chlorine (for non-turbid water) or coagulation/flocculation. Higher performing technologies (i.e. two- or three-stars including membrane filters, UV and coagulants/flocculants) are recommended for vulnerable groups (i.e. those with HIV or young infants) and where the specific pathogen of concern is not known. A list can be found here: <http://www.who.int/household_water/scheme/products/en/> and further information found at the WHO Household Water Treatment site: <http://www.who.int/household_water/scheme/en/>  Drinking water meets WHO *Guidelines for drinking-water quality* (2011) or national standards: <http://www.who.int/water_sanitation_health/publications/dwq-guidelines-4/en/> |  |
| **1.10** | Evidence of documented chlorine residuals should be available from previous testing. |  |
| **1.11** | Drinking water meets WHO *Guidelines for drinking-water quality* (2011) or national standards: <http://www.who.int/water_sanitation_health/publications/dwq-guidelines-4/en/> |  |
| **SANITATION** | | |
| **2.1** | At least four toilets per outpatient setting (one for staff, and for patients: one for females, one for males, one for disabled users). More latrines may be needed depending on the size of the facility. Improved sanitation facilities include flush toilets into managed sewer or septic tank and soakaway pit, VIP latrines, pit latrines with slab, and composting toilets. To be considered usable, a toilet/latrine should have a door which is unlocked when not in use (or for which a key is available at any time) and can be locked from the inside during use, there should be no major holes in the structure, the hole or pit should not be blocked, water should be available for flush/pour flush toilets, and there should be no cracks, or leaks in the toilet structure. It should be within the grounds of the facility and it should be clean as noted by absence of waste, visible dirt and excreta and insects. | |
| **2.4** | Toilets should have a bin for disposal of waste or an area for washing, with water available. | |
| **2.5** | A toilet can be considered to meet the needs of people with reduced mobility if it meets the following conditions: can be accessed without stairs or steps, handrails for support are attached either to the floor or sidewalls, the door is at least 80 cm wide, the toilet has a raised seat (between 40-48cm from the floor), a backrest and the cubicle has space for circulation/manoeuvrings (150x150 cm). The sink tap, and water outside should also be accessible and the top of the sink 75cm from the floor (with knee clearance). Switches for lights, where relevant, should also be at an accessible height (max 120 cm). All specifications are based on ISO 21542:2011 – Building construction - Accessibility and usability of the built environment, available at: http://www.iso.org/iso/home/store/catalogue_tc/catalogue_detail.htm?csnumber=50498 | |
| **2.6** | A functional hand hygiene station may consist of soap and water with a basin/pan for washing hands. **Water should not be chlorinated**. **Alcohol-based hand rub is *not* suitable** for use at latrines. | |
| **2.7** | For low literacy or illiterate cleaners, this should be adapted and simplified accordingly with recognizable pictures and illustrations. | |
| **2.8, 2.9** | No leakage from pipes nor soakaway pit, and soakaway more than 30 m from water source, with grease trap and no visible pool of stagnant water. | |
| **2.10** | Lighting for latrines is necessary in all facilities where night-time services are provided and where there is not sufficient natural light to safely use the latrine during the day. | |
| **HEALTHCARE WASTE** | | |
| **2.12** | Functional means containers should not be ¾ full, be leak-proof with a lid and clearly labelled (i.e. easily distinguishable according to a colour, label or symbol). | |
| **2.15** | Incinerator (if designed for infectious waste and not just general waste) must follow specific design requirements (e.g. use of fire bricks/refractory bricks and mortar (vs. common building bricks) that can withstand the temperatures needed for these incinerators (greater than 800° C). For complete burning, a dual chamber incinerator is needed that reaches temperatures above 800° C and 1100° C, respectively. In case dual incinerators are not available and there is an immediate need for public health protection, small scale incinerators might be used. This involves a compromise between the environmental impacts from controlled combustion with an overriding need to protect public health if the only alternative is indiscriminate dumping. These circumstances exist in many developing situations and small-scale incineration can be a realistic response to an immediate requirement. For guidelines, see WHO (2014) *Safe management of waste from health-care activities.*  Waste may be treated off site. If so, there should be a means to confirm it is treated safely once removed from the facility premises. | |
| **2.18** | Unless a refrigerated storage room is available, storage times for infectious waste (e.g. the time between generation and treatment) should not exceed the following periods:  Temperate climate: 72 hours in winter / 48 hours in summer.  Warm climate: 48 hours during the cool season / 24 hours during the hot season.  Fenced area protected from flooding + lined and covered pit > 30 m from water source + no unprotected healthcare waste is observed. If waste removed offsite, both the site and the holding area (minus the pit) should comply with the above requirements. | |
| **2.19** | Placenta pits: lined or unlined depending on the geology, with slab, with ventilation pipe. | |
| **2.20** | Ash pits: lined or unlined depending on the geology but must prevent leaching to the environment, with slab, bottom of pit at least 1,5 m away from groundwater table. If water gets into the ash pit, it can leach pollutants into the soil. | |
| **2.22** | Protective equipment for people handling waste management includes: gloves, apron and tough rubber boots. | |
| **HAND HYGIENE** | | |
| **3.1** | Point of care is where three elements come together: the patient, the health-care workers and care or treatment involving contact with the patient or his/her surroundings. This may include consultation rooms, operating rooms, delivery rooms AND laboratory. Hand hygiene stations should have a sink or bucket with tap and water with soap OR alcohol-based hand-rub. There should be at least two hand hygiene stations in a ward with more than 20 beds.  Verify that water is available from the tap. | |
| **3.2** | Key places include at points of care, the waiting room, at the facility’s entrance and within 5 m of latrines. | |
| **3.3** | Sink or bucket with tap and water with soap OR alcohol-based hand-rub. Service areas include sterilization, laboratory, kitchen, laundry, showers, waste zone and mortuary. **(Toilets are included under 2.7)** | |
| **3.4** | Tap and water with soap. | |
| **NOTES – ENVIRONMENT, CLEANLINESS AND DISINFECTION (FACILITY ASSESSMENT)** | | |
| **3.8** | Clean as noted by absence of waste, visible dirt and excreta and insects. Environmental surfaces or objects contaminated with blood, other body fluids, secretions or excretions are cleaned and disinfected as soon as possible using standard hospital detergents/disinfectants. | |
| **3.10** | Waste disposal staff who operate the incinerator should have an apron, gloves, goggles, face mask and boots. | |
| **NOTES – MANAGEMENT (FACILITY ASSESSMENT)** | | |
| **4.2** | The budget refers to that used for capital and operational costs. It could be from the community-management group and/or the government, according to the policies and practices in the country. | |

**Facility Assessment - Notes**

**Finish assessing all the wards before you go on to assess the facility. Before you begin you assessment and scoring, take the time to walk through all areas in the facility, including the outside area, the kitchen, the laundry… inspect the incinerator facilities, water supply and management of waste, including grey water… and check availability and count numbers of functional latrines, toilets, lightening, drinking water stations, hand washing stations, soap dispensers, bins, cleaning material, availability of charts and signs in all the areas of the facility other than the wards. Speak to staff, take pictures where needed (no patients/staff/hospital identifying signs allowed in pictures; ONLY THE THREE WASH TEAM SUPERVISORS (i.e. NOT the team leaders) ARE ALLOWED TO TAKE PICTURES). Sit with the team and try to score some of the indicators. If needed walk through the facility again to gather mode details which will allow you to fine-tune the scoring. In the process, write down all your questions. You can then address all your queries with a member of staff in one go. This should allow you to complete the assessment. The assessment refers to the OVERALL scoring for the facility after having observed all wards and all areas.**

**ENTER FACILITY CODE: _____________________________**

| **OVERALL FACILITY ASSESSMENT**  **ENTER FACILITY CODE:_________________** | | **BASELINE ASSESSMENT**  **(Clearly tick the box that best describes your findings)** | | | **Assessment Date:** |
| --- | --- | --- | --- | --- | --- |
| **1** | **WATER** | **Meets**  **Target**  **(+++)** | **Partially Meets Target**  **(++)** | **Does not**  **Meet Target**  **(+)** | **Explain the reasoning for your scoring and comment as appropriate. For indicators with an asterisk, read the notes before scoring.** |
| **1.1*** | Improved water supply piped into the facility or on premises and available. | **□** Yes, improved water supply within facility and available | **□** Improved water supply on premises, (outside of facility building) and available | **□** No improved water source within facility grounds, or improved supply in place but not available. |  |
| **1.2*** | Water services available at all times and of sufficient quantity for all uses (refers to a typical average week) | **□** Yes, every day and of sufficient quantity | **□** More than 5 days per week or every day but not sufficient quantity | **□** Fewer than 5 days per week |  |
| **1.3*** | A reliable drinking water station is present and accessible for staff, patients and carers at all times and in all locations/wards. | **□** Yes, at all times/wards and accessible to all | **□** Sometimes, or only in some places or not available for all users | **□** Not available |  |
| **1.4** | Drinking water is safely stored in a clean bucket/ tank with cover and tap. | **□** Yes | **□** Only some available drinking water points are safely stored | **□** Not safely stored in any water points or no drinking water available |  |
| **1.5** | Sanitary inspection risk score (using Sanitary Inspection Form). | **□** Low risk | **□** Medium risk | **□** High or very high risk |  |
| **1.6** | All end points (i.e. taps) are connected to an available and functioning water supply. | **□** Yes, all are connected and functioning | **□** More than half of all endpoints are connected and functioning | **□** No, less than half of all endpoints connected and functioning |  |
| **1.7** | Water services available throughout the year (i.e. not affected by seasonality, climate change-related extreme events or other constraints). Refers to ‘in a typical average year’. | **□** Yes, throughout the year | **□** Water shortages for 1-2 months | **□** Water shortages for 3 months or more |  |
| **1.8*** | Water storage is sufficient to meet the needs of the facility for 2 days. | **□** Yes | **□** More than 75% of needs met | **□** Less than 75% of needs met |  |
| **1.9*** | Water is treated and collected for drinking with a proven technology that meets WHO performance standards. | **□** Yes | **□** Treated but not regularly | **□** Not treated |  |
| **1.10*** | Drinking water has appropriate chlorine residual (0.2mg/l or 0.5mg/l in emergencies) or 0 *E.coli/100 ml* and is not turbid. | **□** Yes | **□** Chlorine residual exists but is <0.2mg/l | **□** Not treated / do not know residual /do not have capacity to test residual/ no drinking water available |  |
| **1.11*** | National water quality standards exist, and the facility water supply is regulated according to these standards. | **□** Yes, and water meets national standards. | **□** Yes, but no regulation or testing. | **□** No standards exist. |  |
| **1.12** | Sufficient energy is available for pumping and boiling water (mark if not applicable). | **□** Yes, always | **□** Yes, sometimes | **□** Never |  |
| **1.13** | At least one shower or bathing area is available per 40 patients in inpatient settings and is functioning and accessible. | **□** Yes | **□** Showers available, but no water or in disrepair or showers available but fewer than1 per 40 | **□** No showers. |  |
| **1.14** | Shower(s) are adequately lit, including at night. | **□** Yes | **□** Lighting infrastructure exists, but not functioning | **□** Not adequately lit or no lighting infrastructure |  |

| **OVERALL FACILITY ASSESSMENT**  **ENTER FACILITY CODE:_________________** | | **BASELINE ASSESSMENT**  **(Clearly tick the box that best describes your findings)** | | | **Assessment Date:** |
| --- | --- | --- | --- | --- | --- |
| **2.a** | **SANITATION** | **Meets**  **Target**  **(+++)** | **Partially Meets Target**  **(++)** | **Does not**  **Meet Target**  **(+)** | **Explain the reasoning for your scoring and comment as appropriate. For indicators with an asterisk, read the notes before scoring.** |
| **2.1*** | Number of available and usable toilets or improved latrines for patients. | **□** 4 or more (outpatients) and one per 20 users (inpatients). | **□** Sufficient number present but not all functioning or insufficient number. | **□** Less than 50% of required number of latrines available and functioning. |  |
| **2.2** | Toilets or improved latrines clearly separated for staff and patients and visitors. | **□** Yes. | **□** Some separate latrines but not for all three categories (staff, patients and visitors). | **□** No separate latrines. |  |
| **2.3** | Toilets or improved latrines clearly separated for male and female. | **□** Yes. | N/A. | **□** No indication of gender separation. |  |
| **2.4*** | At least one toilet or improved latrine provides the means to manage menstrual hygiene needs. | **□** Yes. | **□** Yes, but toilet is not clean or in disrepair. | **□** No. |  |
| **2.5*** | At least one toilet meets the needs of people with reduced mobility. | **□** Yes. | **□** Yes, but not available or in disrepair. | **□** No toilets for disabled users. |  |
| **2.6*** | Functioning hand hygiene stations within 5 m of latrines. | **□** Yes. | **□** Present, not functioning or no water or soap. | **□** Not present. |  |
| **2.7*** | Record of cleaning visible and signed by the cleaners each day. | **□** Yes. | **□** Toilets cleaned but not recorded. | **□** No record / toilets cleaned less than once a day. |  |
| **2.8*** | Wastewater is safely managed through use of on-site treatment (i.e. septic tank followed by drainage pit) or sent to a functioning sewer system. | **□** Yes. | **□** Present but not functioning. | **□** Not present. |  |
| **2.9*** | Greywater (i.e. rainwater or wash water) drainage system is in place that diverts water away from the facility (i.e. no standing water) and also protects nearby households. | **□** Yes. | **□** Yes, but not functioning and obvious pools of water. | **□** Not present. |  |
| **2.10*** | Latrines are adequately lit, including at night. | **□** Yes | **□** Lighting infrastructure exists, but not functioning. | **□** Not adequately lit or no lighting infrastructure. |  |

| **OVERALL FACILITY ASSESSMENT**  **ENTER FACILITY CODE:_________________** | | **BASELINE ASSESSMENT**  **(Clearly tick the box that best describes your findings)** | | | **Assessment Date:** |
| --- | --- | --- | --- | --- | --- |
| **2.b** | **HEALTHCARE WASTE** | **Meets**  **Target**  **(+++)** | **Partially Meets Target**  **(++)** | **Does not**  **Meet Target**  **(+)** | **Explain the reasoning for your scoring and comment as appropriate. For indicators with an asterisk, read the notes before scoring.** |
| **2.11** | A trained person is responsible for the management of healthcare waste in the healthcare facility. | **□** Yes, presented and adequately trained. | **□** Appointed but not trained. | **□** Not appointed. |  |
| **2.12*** | Functional waste collection containers for 1) non-infectious (general) waste, 2) infectious waste and 3) sharps waste in close proximity to all waste generation points. | **□** Yes. | **□** Separate bins present but lids missing or more than ¾ full; only two bins (instead of three); or at some but not all waste generation points. | **□** No bins or separate sharps disposal. |  |
| **2.13** | Waste correctly segregated at all waste generation points. | **□** Yes. | **□** Some sorting but not all correctly or not practiced throughout the facility. | **□** No sorting. |  |
| **2.14** | Functional burial pit/fenced waste dump or municipal pick-up available for disposal of non-infectious (non-hazardous/general waste). | **□** Yes. | **□** Pit in facility but insufficient dimensions; overfilled or not fenced and locked; irregular municipal waste pick up, etc. | **□** No pit or other disposal method used. |  |
| **2.15*** | **□** Incinerator or alternative treatment technology for the treatment of infectious and sharp waste is functional and of a sufficient capacity. | **□** Yes. | **□** Present but not functional and/or of a sufficient capacity. | **□** None present. |  |
| **2.16** | Sufficient energy available for incineration or alternative treatment technologies (mark if not applicable) | **□** Yes, always. | **□** Yes, sometimes. | **□** Never. |  |
| **2.17** | Hazardous and non-hazardous waste are stored separately before being treated/disposed of or moved off site. | **□** Yes, separated storage areas available. | **□** Separated storage areas are available but with insufficient capacity or overfilled. | **□** No separated storage areas available. |  |
| **2.18*** | All infectious waste is stored in a protected area before treatment, for no longer than the default and safe time. | **□** Yes. | **□** Treated between 24-48 hours. | **□** Treated after 48 hours or not treated at all. |  |
| **2.19*** | Anatomical- pathological waste is put in a dedicated pathological waste/placenta pit, burnt in a crematory or buried in a cemetery. (mark if not applicable). | **□** Yes. | **□** Pit is present but not used or functional or overfilled or not fenced and locked. | **□** None present. |  |
| **2.20*** | Dedicated ash pits available for disposal of incineration ash (mark if not applicable). | **□** Yes. | **□** Present but not functional or overfilled or not fenced and locked. | **□** None present. |  |
| **2.21** | Protocol or SOP (Standard Operating Procedure) for safe management of healthcare waste clearly visible and legible. | **□** Yes, visible and implemented | **□** Written but not visible or implemented. | **□** No protocol/SOP in place. |  |
| **2.22*** | Appropriate protective equipment for all staff in charge of waste treatment and disposal. | **□** Yes. | **□** Some equipment available, but not for all staff, or available but damaged. | **□** None available. |  |

| **OVERALL FACILITY ASSESSMENT**  **ENTER FACILITY CODE:_________________** | | **BASELINE ASSESSMENT**  **(Clearly tick the box that best describes your findings)** | | | **Assessment Date:** |
| --- | --- | --- | --- | --- | --- |
| **3.a** | **HAND HYGIENE** | **Meets**  **Target**  **(+++)** | **Partially Meets Target**  **(++)** | **Does not**  **Meet Target**  **(+)** | **Explain the reasoning for your scoring and comment as appropriate. For indicators with an asterisk, read the notes before scoring.** |
| **3.1*** | Functioning hand hygiene stations are available at all points of care. | **□** Yes. | **□** Stations present, but no water and/or soap or alcohol hand-rub solution. | **□** Not present. |  |
| **3.2*** | Hand hygiene promotion materials clearly visible and understandable at key places. | **□** Yes. | **□** Some places but not all. | **□** None. |  |
| **3.3*** | Functioning hand hygiene stations are available in-service areas. | **□** Yes. | **□** Stations present, but no water and/or soap or alcohol hand-rub solution. | **□** Not present. |  |
| **3.4*** | Functioning hand hygiene stations available in waste disposal area | **□** Yes. | **□** Stations present, but no water and/or soap. | **□** Not present. |  |
| **3.5** | Hand hygiene compliance activities are undertaken regularly. | **□** Yes. | **□** Compliance activities in policy, but not carried out with any regularity. | **□** No compliance activities. |  |

| **OVERALL FACILITY ASSESSMENT**  **ENTER FACILITY CODE:_________________** | | **BASELINE ASSESSMENT**  **(Clearly tick the box that best describes your findings)** | | | | **Assessment Date:** |
| --- | --- | --- | --- | --- | --- | --- |
| **3.b** | **ENVIRONMENT, CLEANLINESS AND DISINFECTION** | **Meets**  **Target**  **(+++)** | | **Partially Meets Target**  **(++)** | **Does not**  **Meet Target**  **(+)** | **Explain the reasoning for your scoring and comment as appropriate. For indicators with an asterisk, read the notes before scoring.** |
| **3.6** | The exterior of the facility is well-fenced, kept generally clean (free from solid waste, stagnant water, no animal and human faeces in or around the facility premises, etc.). | **□** Yes. | | **□** Partly but improvements could be made. Yes, sometimes. | **□** Not kept clean at all. |  |
| **3.7** | General lighting sufficiently powered and adequate to ensure safe provision of healthcare including at night (mark if not applicable). | **□** Yes, always. | | **□** Yes, sometimes. | **□** Never. |  |
| **3.8*** | Floors and horizontal work surfaces appear clean. | **□** Yes. | | **□** Some floors and work surfaces appear clean, but others do not. | **□** Most and/or all floors and surfaces are visibly dirty. |  |
| **3.9** | Appropriate and well-maintained materials for cleaning (i.e. detergent, mops, buckets, etc.) are available. | **□** Yes. | | **□** Yes, available but not well maintained. | **□** No materials available. |  |
| **3.10*** | At least two pairs of household cleaning gloves and one pair of overalls or apron and boots in a good state, for each cleaning and waste disposal staff member. | **□** Yes. | | **□** Available but in poor condition. | **□** Not available. |  |
| **3.11** | At least one member of staff can demonstrate the correct procedures for cleaning and disinfection and apply them as required to maintain clean and safe rooms. | **□** Yes. | | **□** Procedure is known but not applied. | **□** Procedure not known or applied. |  |
| **3.12** | Beds have insecticide treated nets to protect patients from mosquito-borne diseases. | **□** Yes, on all beds. | | **□** Available on some but not all beds, or available but with rips and or holes. | **□** No bed nets available. |  |
| **3.13** | A mechanism exists to track supply of IPC-related materials (such as gloves and protective equipment) to identify stock-outs. | **□** Yes. | | **□** Mechanism exists but is not enforced. | **□** No mechanism exists. |  |
| **3.14** | Record of cleaning visible and signed by the cleaners each day. | **□** Yes. | | **□** Record exists but is not completed daily or is outdated. | **□** No record of floors and surfaces being cleaned. |  |
| **3.15** | Laundry facilities are available to wash linen from patient beds between each patient. | **□** Yes. | | **□** Facilities exist but are not working or not being used. | **□** No facilities, and/or no linen. |  |
| **3.16** | The facility has sufficient natural ventilation and where the climate allows, large opening windows, skylights and other vents to optimize natural ventilation. | **□** Yes. | | **□** Some ventilation but not well maintained or insufficient to produce natural ventilation. | **□** No. |  |
| **3.17** | Kitchen stores and prepared food is protected from flies, other insects or rats. | **□** Yes. | | N/A | **□** No. |  |
| **3.18** | Beds for patients should be separated by a distance of 2.5 metres from the centre of one bed to the other and each bed has only one patient. | **□** Yes, all beds meet this guidance. | | **□** Some but not all beds fit this criteria. | **□** No beds meet this criteria. |  |
| **OVERALL FACILITY ASSESSMENT**  **ENTER FACILITY CODE:_________________** | | | **BASELINE ASSESSMENT**  **(Clearly tick the box that best describes your findings)** | | | **Assessment Date:** |
| **4** | **MANAGEMENT** | | **Meets**  **Target**  **(+++)** | **Partially Meets Target**  **(++)** | **Does not**  **Meet Target**  **(+)** | **Explain the reasoning for your scoring and comment as appropriate. For indicators with an asterisk, read the notes before scoring.** |
| **4.1** | WASH FIT or other quality improvement/management plan for the facility is in place, implemented and regularly monitored. | | **□** Yes. | **□** Complete but has not been implemented and/or is not monitored, or incomplete. | **□** No plan. |  |
| **4.2*** | An annual planned budget for the facility is available and includes funding for WASH infrastructure, services, personnel and the continuous procurement of WASH items (hand hygiene products, minor supplies to repair pipes, toilets, etc.) which is enough to meet the needs of the facility. | | **□** Yes. | **□** Yes, but budget is insufficient. | **□** No budget. |  |
| **4.3** | An up-to-date diagram of the facility management structure is clearly visible and legible. | | **□** Yes. | **□** Yes, but not up to date. | **□** Not available. |  |
| **4.4** | Adequate cleaners and WASH maintenance staff are available. | | **□** Yes. | **□** Some available, but not adequate or not skilled/ motivated. | **□** None available. |  |
| **4.5** | A protocol for operation and maintenance, including procurement of WASH supplies is visible, legible and implemented. | | **□** Yes. | **□** Protocol exists but not implemented. | **□** No protocol. |  |
| **4.6*** | Regular ward-based audits are undertaken to assess the availability of hand-rub, soap, single use towels and other hand hygiene resources. | | **□** Yes. | **□** Undertaken less than once a week or assessment is incomplete. | **□** Not undertaken. |  |
| **4.7** | New healthcare personnel receive IPC training as part of their orientation program. | | **□** Yes. | **□** Some but not all staff. | **□** No training. |  |
| **4.8** | Healthcare staff are trained on WASH/IPC each year. | | **□** Yes. | **□** Staff are trained but not every year or only some staff are trained. | **□** No training. |  |
| **4.9** | Facility has a dedicated WASH or IPC focal person. | | **□** Yes. | **□** Yes, but focal point does not have sufficient time, resources or motivation to carry out duties. | **□** No. |  |
| **4.10** | All staff have a job description written clearly and legibly, including WASH-related responsibilities and are regularly appraised on their performance. | | **□** Yes. | **□** Some, but not all, staff have a job description, or their performance is not appraised. | **□** No job description written. |  |
| **4.11** | High performing staff are recognized and rewarded and those that do not perform are dealt with accordingly. | | **□** Yes. | **□** Either high or low performers addressed but not both. | **□** No action or recognition of staff based on performance. |  |

**Sanitary Inspection Form - Notes**

**Do your assessment with the public health officer on site. Always write the name of the company supplying water to the facility (where applicable).**

**ENTER FACILITY CODE: _____________________________**

**SANITARY INSPECTION FORM 1**: DUG WELL WITH HAND PUMP

**I. General information**

1. Code of facility:
2. Location and/or name of dug well:
3. Date of inspection:
4. Weather conditions during inspection:

*Note. If there is more than one dug well accessed by the facility, or if the facility uses other water sources (such as springs or boreholes), carry out sanitary inspections for these sources too.*

**II. Specific questions for assessment**

1. Is the source located at an unsafe distance from an unsealed latrine (i.e. a latrine near is uphill or at a location where the groundwater gradient would flow from the latrine to the water source)? Y/N
2. Is the fence absent, inadequate or faulty? Y/N
3. Can animals have access within 30 metres (100 feet) of the well? Y/N
4. Is there any other source of pollution within 30 metres (100 feet) of the well
   (such as animal breeding, farming, roads, healthcare waste, domestic garbage)? Y/N
5. Is there stagnant water within 3 metres of the well? Y/N
6. Is the drainage channel absent or cracked, broken or in need of cleaning? Y/N
7. Is the cement floor or slab less than 2 metres in diameter around the top of the well? Y/N
8. Are there cracks in the cement floor or slab? Y/N
9. Is the hand pump loose at the point of attachment or, for rope-washer pumps,
   is the pump cover missing or damaged? Y/N
10. Is the well cover absent, cracked or insanitary? Y/N

Total score of risk factors as total number of “YES” answers:

**III. Results and comments**

1. Sanitary inspection risk score (*tick appropriate box*):

| **Very high risk** (risk score: 9–10) | **High risk** (risk score: 6–8) | **Medium risk** (risk score: 3–5) | **Low risk** (risk score: 0–2) |
| --- | --- | --- | --- |
|  |  |  |  |

1. Important points of risk noted and reported on the reverse of this form:

- list according to question numbers 1–10
- additional comments.

**IV. Names and signatures of assessors: ……………………………………………………………….**

**SANITARY INSPECTION FORM 2**: DEEP BOREHOLE WITH MOTORIZED PUMP

**I. General information**

1. Code of facility:
2. Location and/or name of borehole:
3. Date of inspection:
4. Weather conditions during inspection:

*Note. If there is more than one borehole accessed by the facility, or if the facility uses other water sources (such as springs or dug wells), carry out sanitary inspections for these sources too.*

**II. Specific questions for assessment**

1. Is there a latrine or sewer within 15–20 m of the extraction site/well-head? Y/N

2. Is the nearest latrine a pit latrine that percolates to soil, i.e. not connected to a septic

tank or sewer? Y/N

3. Is there any other source of pollution (e.g. animal excreta, rubbish, surface water)

within 10 m of the borehole? Y/N

4. Is there an uncapped well within 15–20 m of the borehole? Y/N

5. Is the drainage area around the pump house faulty? Y/N

6. Is the fencing around the installation damaged in any way which would permit any

unauthorized entry or allow animals access? Y/N

7. Is the floor of the pump house permeable to water? Y/N

8. Is the well seal unsanitary? Y/N

9. Is the chlorination functioning properly? Y/N

10. Is chlorine present at the sampling tap? Y/N

Total score of risk factors as total number of “YES” answers:

**III. Results and comments**

- 1. Sanitary inspection risk score (*tick appropriate box*):

| **Very high risk** (risk score: 9–10) | **High risk** (risk score: 6–8) | **Medium risk** (risk score: 3–5) | **Low risk** (risk score: 0–2) |
| --- | --- | --- | --- |
|  |  |  |  |

- 1. Important points of risk noted and reported on the reverse of this form:
- list according to question numbers 1–10
- additional comments.

**IV. Names and signatures of assessors: ……………………………………………………………….**

**SANITARY INSPECTION FORM 3**: PUBLIC/YARD TAPS AND PIPED DISTRIBUTION

**I. General information**

1. Code of facility:
2. Date of inspection:
3. Weather conditions during inspection:
4. Location and/or name of water source(s) feeding the distribution system:
5. Location and/or name of storage reservoir feeding the distribution system (if any):

*Note. If the distribution system is served by a storage reservoir, also carry out a sanitary inspection using the form “Storage reservoirs”.*

**II. Specific questions for assessment**

*Note. Fill in one form per public or yard tap under inspection. In facilities with water piped directly into the building only questions 7–10 apply. Not all taps within the facility need to be inspected in every inspection round; a selected sample is enough.*

*Public or yard tap*

1. Does the tap leak? Y/N
2. Is the tap or are attachments (such as hoses) insanitary? Y/N
3. Does spilt water accumulate around the tap stand? Y/N
4. Is the area around the tap stand polluted by waste, faeces or other materials? Y/N
5. Is the area around the tap stand unfenced, allowing animals to access the area? Y/N
6. Is there a sewer or a latrine at an unsafe distance from the tap stand (generally 30 m but may be more or less depending on the gradient, geology and size of water or sewer infrastructure) Y/N

*Piped distribution*

1. Are there any signs of leaks in the inspection area (for example, accumulating water)? Y/N
2. Are any of the pipes exposed above ground in the inspection area? Y/N
3. Have users report any pipe breaks within the last week? Y/N
4. Has there been discontinuity in the last 10 days? Y/N

Total score of risk factors as total number of “YES” answers:

**III. Results and comments**

1. Sanitary inspection risk score (*tick appropriate box*):

*Note. In situations where, only questions 6–10 apply, the score below can be adapted as follows: “Very high” = 5; “High” = 3–4; “Medium” = 2; “Low” = 0–1.*

| **Very high risk** (risk score: 9–10) | **High risk** (risk score: 6–8) | **Medium risk** (risk score: 3–5) | **Low risk** (risk score: 0–2) |
| --- | --- | --- | --- |
|  |  |  |  |

1. Important points of risk noted and reported on the reverse of this form:

- list according to question numbers 1–10
- additional comments.

**IV. Names and signatures of assessors: ……………………………………………………………….**

**SANITARY INSPECTION FORM 4**: RAINWATER HARVESTING

**I. General information**

1. Code of facility:
2. Location and/or name of rainwater storage:
3. Date of inspection:
4. Weather conditions during inspection:

*Note. If the facility uses other water sources (such as springs or boreholes), carry out sanitary inspections for these sources too.*

**II. Specific questions for assessment**

1. Is there any visible contamination of the roof catchment area (plants, dirt, or excreta)? Y/N

2. Are the guttering channels that collect water dirty? Y/N

3. Is there any deficiency in the filter box at the tank inlet (e.g. lacks fine gravel)? Y/N

4. Is there any other point of entry to the tank that is not properly covered? Y/N

5. Is there any defect in the walls or top of the tank (e.g. cracks) that could let water in? Y/N

6. Is the tap leaking or otherwise defective? Y/N

7. Is the concrete floor under the tap defective or dirty? Y/N

8. Is the water collection area inadequately drained? Y/N

9. Is there any source of pollution around the tank or water collection area (e.g. excreta)? Y/N

10. Is a bucket in use and left in a place where it may become contaminated? Y/N

Total score of risk factors as total number of “YES” answers:

**III. Results and comments**

- 1. Sanitary inspection risk score (*tick appropriate box*):

| **Very high risk** (risk score: 9–10) | **High risk** (risk score: 6–8) | **Medium risk** (risk score: 3–5) | **Low risk** (risk score: 0–2) |
| --- | --- | --- | --- |
|  |  |  |  |

- 1. Important points of risk noted and reported on the reverse of this form:
- list according to question numbers 1–10
- additional comments.

**IV. Names and signatures of assessors: ……………………………………………………………….**

**SANITARY INSPECTION FORM 5**: STORAGE RESERVOIRS

**I. General information**

1. Code of facility:
2. Location and/or name of storage reservoir:
3. Date of inspection:
4. Weather conditions during inspection:
5. Location and/or name of water source(s) feeding the reservoir:

*Note. If there is more than one storage reservoir used in your facility, use one form for each reservoir.*

*Note. If the storage reservoir feeds a piped distribution system, also carry out a sanitary inspection using the form “Public/yard taps and piped distribution”.*

*Note. If the storage reservoir is equipped with a tap for collecting water, also carry out a sanitary inspection using questions 1–5 of the form “Public/yard taps and piped distribution”.*

**II. Specific questions for assessment**

1. Is there any point of leakage of the pipe between source and storage reservoir? Y/N
2. Is the physical infrastructure of the storage reservoir cracked or leaking? Y/N
3. Is the inspection cover of the storage reservoir absent or open? Y/N
4. Is the inspection cover faulty, corroded or is the concrete around the cover damaged? Y/N
5. Is the inspection cover visibly dirty? Y/N
6. Are screens protecting the air vents on the storage reservoir missing or damaged? Y/N
7. If there is an overflow pipe, is the screen protecting it missing or damaged? Y/N
8. Is there any scum or foreign object in the storage reservoir? Y/N
9. Is the diversion ditch above the storage reservoir absent or non-functional? Y/N
10. Is the area around the storage reservoir unfenced or is the fence damaged, allowing animals to access the area? Y/N
11. Is the storage reservoir not regularly cleaned and disinfected? Y/N

Total score of risk factors as total number of “YES” answers:

**III. Results and comments**

1. Sanitary inspection risk score (*tick appropriate box*):

| **Very high risk** (risk score: 9–11) | **High risk** (risk score: 6–8) | **Medium risk** (risk score: 3–5) | **Low risk** (risk score: 0–2) |
| --- | --- | --- | --- |
|  |  |  |  |

1. Important points of risk noted and reported on the reverse of this form:

- list according to question numbers 1–11
- additional comments.

**IV. Names and signatures of assessors:…………………………………………………………..**
