# Supplementary material for: Evaluating the foundations that help avert antimicrobial resistance: Performance of essential water sanitation and hygiene functions in hospitals and requirements for action in Kenya
Source: PLoS One. 2019 Oct 9;14(10):e0222922. doi: 10.1371/journal.pone.0222922 (PMC6785173; doi:10.1371/journal.pone.0222922)
Supplement: S3 File — (DOCX) [file pone.0222922.s004.docx]

**S 3File**. **Ward level Aggregate by Domain for Water, Hygiene and Organisation Management**


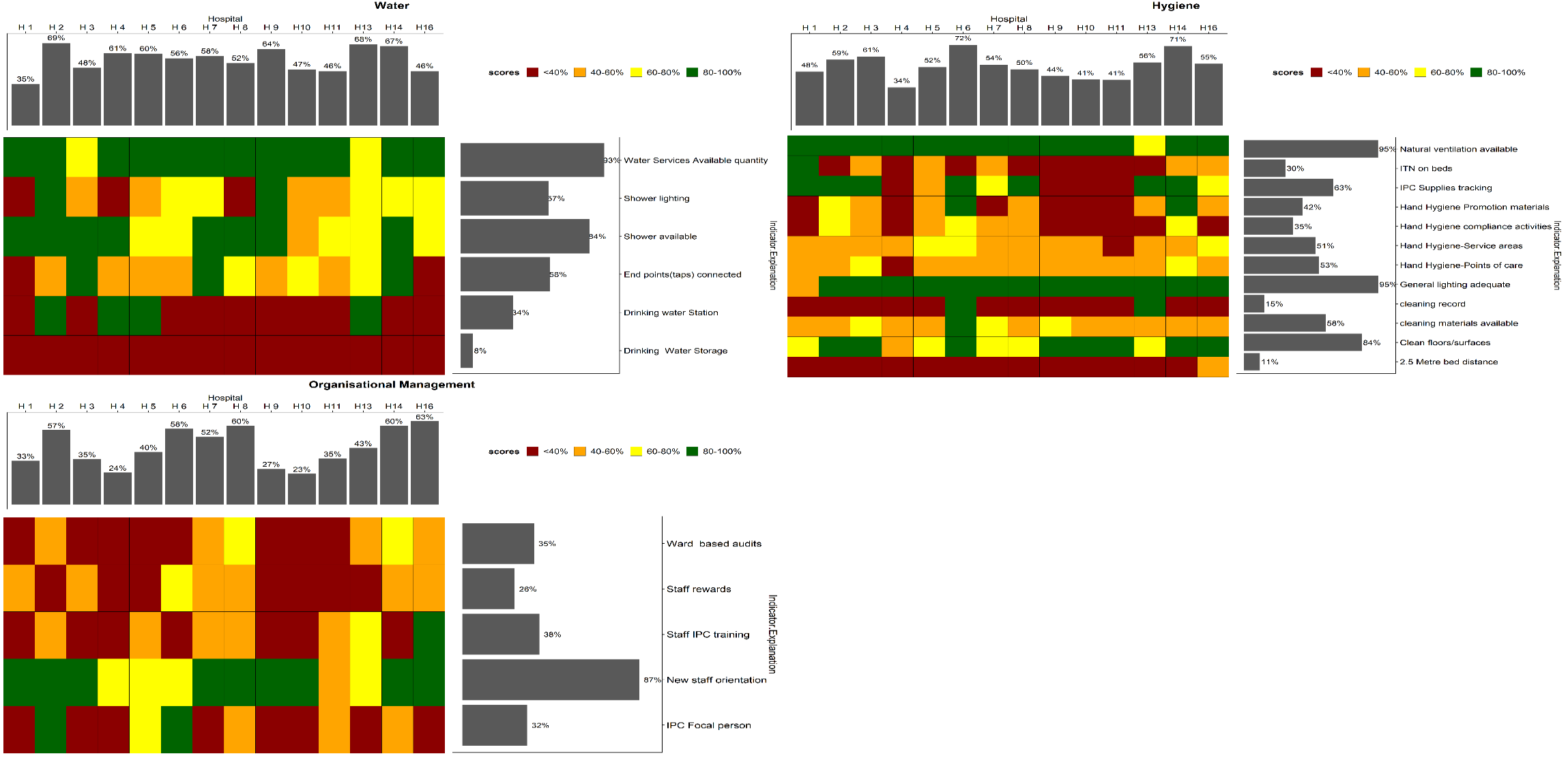


*Multi-panel plot showing the mean service performance at ward level for all the water, hygiene and organisational management domain. The vertical bars indicate the mean ward performance in each hospital. The horizontal summarise the performance of each indicator across all the hospital wards. The squares in the central grid are coloured according to the performance classification of each indicator in each hospital using % cut-offs.*
