# Supplementary material for: Evaluating the foundations that help avert antimicrobial resistance: Performance of essential water sanitation and hygiene functions in hospitals and requirements for action in Kenya
Source: PLoS One. 2019 Oct 9;14(10):e0222922. doi: 10.1371/journal.pone.0222922 (PMC6785173; doi:10.1371/journal.pone.0222922)
Supplement: S4 File — (DOCX) [file pone.0222922.s005.docx]

**S4 File. Performance of Indicators under the infection prevention and control committee and hospital management**


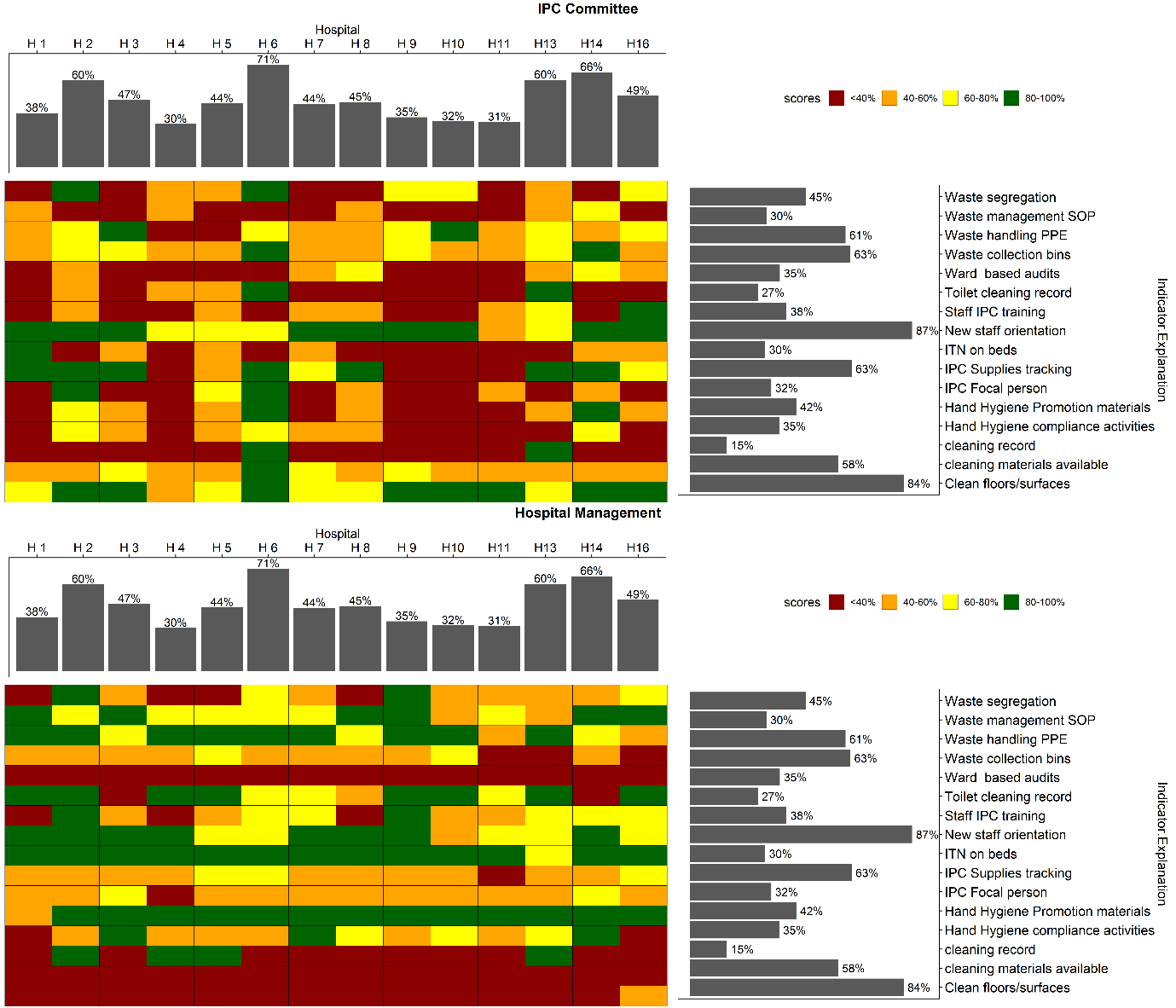


*Shows mean service performance at ward level for indicators under the infection prevention and control and hospital management domains shown by the vertical bars. The horizontal bars summarise the performance of each indicator across all the hospitals. The central tiles in the central grid are coloured according to the performance classification of each indicator in each hospital using % cut-offs.*
