# Supplementary material for: Evaluating the foundations that help avert antimicrobial resistance: Performance of essential water sanitation and hygiene functions in hospitals and requirements for action in Kenya
Source: PLoS One. 2019 Oct 9;14(10):e0222922. doi: 10.1371/journal.pone.0222922 (PMC6785173; doi:10.1371/journal.pone.0222922)
Supplement: Attachment — Submitted filename: Response to Reviewers 30.08.19.docx [file pone.0222922.s006.docx]

# KEMRI/ WELLCOME TRUST RESEARCH PROGRAMME

(Centre for Geographic Medicine Research – Coast)

**P.O.Box 230, 80108, Kilifi, Kenya	P.O.Box 43640, 00100, Nairobi, Kenya**

**Tel: (+254) 417 522535/522063/525044/525453	Tel: (+254) 20 2710672/2715160**

**Fax: (+254) 417 522390	Fax: (+254) 20 2711673**

**Email:** mmaina@kemri-wellcome.org

27^th^ August 2019

To:	The Editor

Plos One, Antimicrobial Resistance for papers

**Re: PONE-D-19-19421: Evaluating the foundations that help avert antimicrobial resistance: Performance of essential water sanitation and hygiene functions in hospitals and requirements for action in Kenya**

Thank you for the review and comments provided for this manuscript. We have made the changes as suggested.

**The Editors comments** :

1. Please ensure that your manuscript meets PLOS ONE's style requirements, including those for file naming.

**Response:**

As per the templates provided, we have made the following changes

**Title, Author and Affiliations**

- Article Title: Both the long and short title are now edited sentence case Page 1 Line 1-5
- Affiliations: These have been modified to include each component from small to large. (Page 1 Line 10-13)

The abbreviation UK has also been written in full( Page 1 Line 8)

- Corresponding Authorship: The initials of the corresponding author have been included (Page 1 Line 15)

**Manuscript Body**

**Abstract**

- Abstract title: Level 1 heading used with bold font 18pt (Page 2 Line 20)
- Other headings in the abstract have been edited to Level 2 headings, bold font 16pt

**Introduction**

- Level 1 heading with font size 18pt used. Heading changed from background to introduction. (Page 3 Line 48)
- Figure 1 citations changed to Fig 1. Captions edited to reflect the journal requirements (Page 5 Line 80)
- Supplemental material renamed appropriately to S1 Table (Page 3 line 85)

**Methods**

- Level 2 and 3 headings edited appropriately to correct fonts in the methods section
- Supplemental material renamed appropriately to S2 File (Page 6 line 110)
- The term COREQ written in full and the Supplemental material renamed appropriately to S3 File (Page 9 line 180-182)

**Results**

- We have edited the headings to level 2 and 3 headings in the results section as appropriate.
- Footnotes added to explain the abbreviations ENT and ICU on Table 1 (Page 12 line 236)
- Figure 2. citations changed to Fig 2. Captions edited to reflect the journal requirements (Page 13 Line 245-248)
- Figure 3. citations changed to Fig 3. Captions edited to reflect the journal requirements (Page 14 Line 266-271)
- Supplemental material renamed appropriately to S4 File (Page 14 line 282)
- Figure 4. citations changed to Fig 4. Captions edited to reflect the journal requirements (Page 15 Line 285-289)
- Supplemental material renamed appropriately to S5 File (Page 16 line 318)

For the discussion, conclusion, acknowledgements, author contributions and references. These headings have been edited to level 1 heading in bold 18pt font.

**Supporting information**

The list of supporting information has been edited per the journal guidelines

Page 29 Line 638-644

**2.Editor Comments**

a) Please provide an amended Funding Statement that declares *all* the funding or sources of support received during this specific study (whether external or internal to your organization) as detailed online in our guide for authors.

b) Please state what role the funders took in the study.  If any authors received a salary from any of your funders, please state which authors and which funder. If the funders had no role, please state: "The funders had no role in study design, data collection and analysis, decision to publish, or preparation of the manuscript."

Please include your amended statements within your cover letter; we will change the online submission form on your behalf.

**Response**

Thank you. We have included all the three sources of funding for this work. We have also indicated that the funders had no role in study design, data collection and analysis, decision to publish, or preparation of the manuscript. The Full Funding Statement is shown below;

*MM, GK, JM, M.Z and OT were supported by funds through a grant from the Economic and Social Research Council ESRCS # ES/P004938/1 awarded to ME. A Senior Research Fellowship awarded to ME by The Wellcome Trust (#097170) supported P.M. MM received additional support from a grant to the Initiative to Develop African Research Leaders (IDeAL) through the DELTAS Africa Initiative [DEL-15-003], an independent funding scheme of the African Academy of Sciences (AAS)'s Alliance for Accelerating Excellence in Science in Africa (AESA) and supported by the New Partnership for Africa's Development Planning and Coordinating Agency (NEPAD Agency) with funding from the Wellcome Trust [107769/Z/10/Z] and the UK government. The funders had no role in study design, data collection and analysis, decision to publish, or preparation of the manuscript.*

**3.Editors Comments**

We note that you have stated that you will provide repository information for your data at acceptance. Should your manuscript be accepted for publication, we will hold it until you provide the relevant accession numbers or DOIs necessary to access your data. If you wish to make changes to your Data Availability statement, please describe these changes in your cover letter, and we will update your Data Availability statement.

**Response**

We have managed to upload the dataset onto a public repository (Harvard Dataverse). The DOI is also provided. DOI Information: <https://doi.org/10.7910/DVN/IJUWWR>. The data availability statement in the manuscript has been edited appropriately as shown (Page 27 line 584-586)

*All summary data underlying the findings are freely available in the manuscript and supplemental files. The raw data used for this manuscript are hosted in a public repository Harvard Dataverse.* *DOI Information:* [*https://doi.org/10.7910/DVN/IJUWWR*](https://doi.org/10.7910/DVN/IJUWWR)

**Editors Comments**

Thank you for including your ethics statement on the online submission form: "We made every effort to ensure the quality and integrity of the research. We sought and received informed consent in all cases where this was relevant.

We respected the confidentiality and anonymity of our research respondents and checked they were willing to participate in the study voluntarily.

There was no potential for physical harm, but we made every effort to anonymise quotes even though the work is not particularly contentious. We believe the research to be independent and impartial and welcome any further questions.

APPROVALS:

OXTREC approval from the University of Oxford was granted on June 15th, 2017. Ref: 525-17

Approval from the Kenyan Medical Research Institute granted, May 2nd 2017. Ref: KEMRI/SERU/CGMR-C//086/3450". To help ensure that the wording of your manuscript is suitable for publication, would you please also add this statement at the beginning of the Methods section of your manuscript file.

**Response**

Thank you for the comment. We have included an ethics statement at the beginning of the methods section as suggested. Page 6 line 97-104

Thank You

Dr Michuki Maina

On behalf of the Authors
